# Nor weak ties, nor strong ties: Personal support networks and relations between autistic peers

**DOI:** 10.1177/13623613251369908

**Published:** 2025-09-09

**Authors:** Isabelle Courcy, Noémie Cusson, Nuria Jeanneret

**Affiliations:** 1 University of Montreal, Canada; 2 Université du Québec à Montréal, Canada

**Keywords:** adults, autism spectrum disorders, family functioning and support, qualitative research

## Abstract

The aim of this study is to analyze egocentric social networks of 31 autistic adults in Québec (Canada). We examined the composition of their proximal personal support network and the role of support shared among autistic peers. A mixed method analysis was used. An egocentric network questionnaire that included qualitative prompts was conducted to gather participants’ interpretations and reference systems, facilitating an understanding of networks’ multidimensional aspect. A reflexive thematic analysis was conducted with a subset of the data from the qualitative prompts. Three main themes were identified: (1) interference in social interactions, (2) mutual acknowledgment, and (3) social engagement in the community. Most participants (*n* = 21) mentioned autistic peers, but although these peers may have provided support, participants rarely identified them as being part of their proximal personal support network. Results nevertheless highlight the importance of peer support for several participants, which can refer to the concept of “weak ties,” but they differ from it by the exchanges of emotional support, which are normally expected from “strong” ties.

**Lay abstract:**

Social support is recognized as an important predictor of quality of life in autistic and neurodivergent people. However, few studies have explored in detail the composition of support networks in autistic adults. Moreover, research on social networks in the field of autism has mainly focused on the support networks of experts and parents. This article presents the results of a study that analyzed the composition of the support network of 31 autistic adults and examined the role of autistic peer support in their network. Interviews were conducted with the participants. Most of them (*n* = 21) mentioned autistic peers in their social network. Although these peers provided unique types of support, participants rarely identified them as close friends or relatives. Nevertheless, the results underline the importance of peer support for participants, which often presented itself as empathetic listening, the sharing of advice to deal with everyday life issues and company for recreational activities. This study shows that we should not underestimate the support that can be provided by people who share a common experience, even if they are not considered close friends. It paves the way to thinking about how communities and professionals, such as social workers and educators, can support opportunities and facilitate spaces that foster peer support.

Social support is recognized as an important predictor of quality of life of autistic^1^ people (Jeanneret et al., 2022; Jennes-Coussens et al., 2006; Kamio et al., 2013). It facilitates social participation by reducing isolation and loneliness (Tobin et al., 2014). Conversely, lack of social support is associated with higher rates of depression and suicidal thoughts (Hedley et al., 2018). However, few studies have explored in detail the composition of autistic adults’ support networks (Lei et al., 2025), and most compare autistic people’s personal networks with those of allistic^2^ or nonautistic people. Indeed, autism research on social networks has mainly considered expert (e.g. Eyal, 2013) and parent support networks (e.g. Benson, 2012; Courcy & des Rivières-Pigeon, 2021; Laslo-Roth et al., 2023).

According to Lei, Ashwin, et al. (2020) and van Asselt-Goverts et al. (2015), autistic people’s social networks, on average, are significantly smaller than those of allistic people of the same age. Their networks mainly consist of friends and family and, to a lesser extent, figures such as healthcare professionals and social workers (Jeanneret et al., 2022; Lei, Ashwin, et al., 2020; McGhee Hassrick et al., 2020). Lei, Ashwin, et al. (2020) found that 36% of the support networks of autistic students entering university comprised family members and 45% of their networks were made up of friends, similar to their findings on allistic students’ support networks (38%, for family only). In a subsequent longitudinal study, these authors show that the percentage of autistic students’ networks comprising friends increased over time (49%–59%) whereas it remained fairly stable over time for family members (Lei, Brosnan, et al., 2020). Autistic people have known the members of their social network for as long as allistic people but are generally less satisfied (van Asselt-Goverts et al., 2015). Some desire relationships of higher quality, larger networks, and more support to improve their social skills (van Asselt-Goverts et al., 2015; van den Heuvel et al., 2022). Differences have also been noted concerning gender: The networks of women and gender-nonconforming people have more community members (defined as actors who are neither family members nor professionals) compared with those of men (McGhee Hassrick et al., 2020). However, men seem to have more interconnected actors in their networks (McGhee Hassrick et al., 2020).

Social support can improve autistic people’s quality of life, as evidenced by the development of autistic peer support groups (Martin et al., 2017). In an ethnographic study, Ryan Idriss (2021) notes that the support group acts as a social infrastructure in which members can socialize as well as give and receive different types of support based on each other’s needs. These needs generally were not fulfilled elsewhere. Support groups can also be seen as opportunities to make friends and places where one can be oneself without the fear of being judged (Chan et al., 2023). Other studies show that these groups help participants discuss their experiences, share information and practical solutions for overcoming everyday life problems, and reflect on autism and what it means to them personally (Crane et al., 2021; Crompton et al., 2022; Jantz, 2011). They provide fertile ground for the development of a sense of belonging to the autistic community and contribute to the formation of a more positive personal representation of autism (Crane et al., 2021; Crompton et al., 2022).

These studies suggest interesting avenues toward obtaining a better understanding of social support among autistic peers. However, several questions remain understudied, including the following: What types of support are given among autistic peers? In what relational contexts? How important are they to participants in their daily lives? Does autistic peer support differ from support exchanged with allistic family members and friends?

This article presents the results of a study on personal networks conducted with 31 autistic adults in Québec (Canada). The study had two objectives: first, to analyze the composition of participants’ personal support network (ties, type of support, and autistic peer support), and second, to better understand the subjective perceptions that participants have about autistic peer support and the meaning they attribute to these relationships in their personal network.

## Network analysis and the strength of ties

The personal support network includes all the stable social ties a person (ego) maintains, based on the regular sharing of resources, help, or support (Cohen et al., 2000; Haber et al., 2007; Wellman, 1988). Its composition modulates the quality and quantity of support the person has access to (Stewart & Langille, 2000). Moreover, the availability of support does not necessarily mean that the person’s need for support is being met (Song et al., 2014), and conflicts related to unmet expectations can arise (Andersson & Monin, 2018). Several indicators are suggested to analyze the dynamics and processes at work in the social world. Network’s size or density can reflect cohesion, solidarity, or constraints as conditions of possibility for interconnected actors (Marin & Wellman, 2014). Another characteristic is the strength of ties, which refers to a general sense of closeness: Stronger ties correspond to friends, dependable sources of social or emotional support, and weaker ties to acquaintances. Now a classic, Granovetter’s (1973) work shows how “weak ties” have an important role as connectors and facilitate the interpersonal circulation of useful information. These ties are more likely to provide new information and opportunities to ego because they can act as bridges and offer access to new social circles. Stronger ties, such as those with family members or close friends, are likely to provide support to ego.

## Methods

This study is based on pragmatism as a philosophical position that gives priority to the practical consequences and concrete applications of knowledge and research (Abbott, 2004). This pragmatist stance supports the use of different research methods and posits the research process as a continuous cycle of inductive, deductive, and abductive reasoning, thus producing useful knowledge and pursuing a rigorous research aim (Mitchell, 2018). The research design combines quantitative (“number”) and qualitative (“words”) data collection and analysis methods (Denzin & Lincoln, 2018). This strategy was chosen to provide a reliable, rigorous, and more complete description of support networks as well as a better understanding of the role of peer support in these networks based on people’s subjective perceptions. It can be qualified as “mixed methods data analyses” (Onwuegbuzie & Teddlie, 2003 in Creswell & Plano Clark, 2006, p. 11) or a type of mixed methods study “in gray areas” (Creswell & Plano Clark, 2006, p. 11). According to Crossley et al. (2015), “[n]etwork analysis is a formal analytic approach, focused upon patterns of connection” (Crossley et al., 2015, p. 5). However, stressing the fact that the availability of support does not necessarily mean that the person’s need for support is being met (Song et al., 2014), we wanted to situate participants’ subjective perceptions of peer support within their personal support networks. This strategy was aimed at answering research questions and generating results enriched by this combination of quantitative and qualitative analysis.

This study received approval from a university ethics committee. Participants were recruited through a call for participation posted in groups for autistic people and on Facebook pages for university students. To participate in the study, individuals needed to live in Québec (Canada) and be at least 18 years old. Thirty-one autistic adults were recruited. About half were women (*n* = 17) and the other half identified themselves as men (*n* = 14). Participants were aged between 19 and 67, with a mean age of 35. About half lived in Greater Montréal (*n* = 17), and the rest were spread across six different regions in Québec (Canada). The participants had been diagnosed by a professional, except for one who was undergoing assessment. Thirteen participants were a couple and five were parents. Regarding the highest level of education attained, 10 participants had a high school diploma and thirteen had a diploma of college studies or a vocational diploma. Five had a university degree. Two thirds of participants had a full-time or part-time job (*n* = 19). Nine were pursuing college or university studies.

An egocentric network questionnaire that included qualitative prompts was conducted to gather participants’ interpretations and reference systems, facilitating an understanding of networks’ multidimensional aspect (Hogan et al., 2007; Hollstein, 2014). Egocentric networks are based “on individuals and their immediate social environment. A fundamental tenet is that each person lives in a personal community—partially of their own creation and nearly unique to them—whose composition and structure have consequences” (Perry et al., 2018, p. 25). Egocentric network analysis focuses on the structure of network relationships, but a weakness is that insight into the quality and content of support exchanges is limited (Perry et al., 2018). To address this limitation, we added qualitative prompts to the egocentric questionnaire (see Supplementary Material for more details). This helped to better understand the quality of interactions between autistic egos, and their identified alters. The network generator used was “the main people you have interacted with, talked to, or done activities with over the past six months.” Following that, participants were asked to identify those they considered most significant or important in their daily lives (up to a maximum of 5). These ties constituted the proximal personal support network (PPSN). For each alter, questions were asked about the length of the relationship, frequency of contact, and types of support and help given. A visual aid designed to note the different people in the participants’ network was used. According to Hollstein (2014), “mapping networks is a well-suited means of facilitating the discussion of relationships while it provides a strong stimulus for the production of narratives” (p. 10). Once the visual aid was completed, we asked participants to indicate which people knew each other and whether they were aware of any conflicts between them.

### Community involvement

All authors, one of whom is autistic, are involved in the community and contributed to research, especially on the social experience of neurodivergent/neurodiverse people (e.g. Autistics, ADHDers, on the spectrum, Aspie) and on inclusive and participatory research. Some of the study’s findings were shared and discussed with people from the French-speaking autistic community (Québec, France, Switzerland) at three online conferences, held as part of events organized by community organizations. Their comments have enriched our understanding of the results presented in this article.

### Data analysis

The analysis followed a two-step procedure: first, an analysis of egocentric network data followed by a reflexive thematic qualitative analysis.

### Analysis of egocentric network data

**Figure 1.**
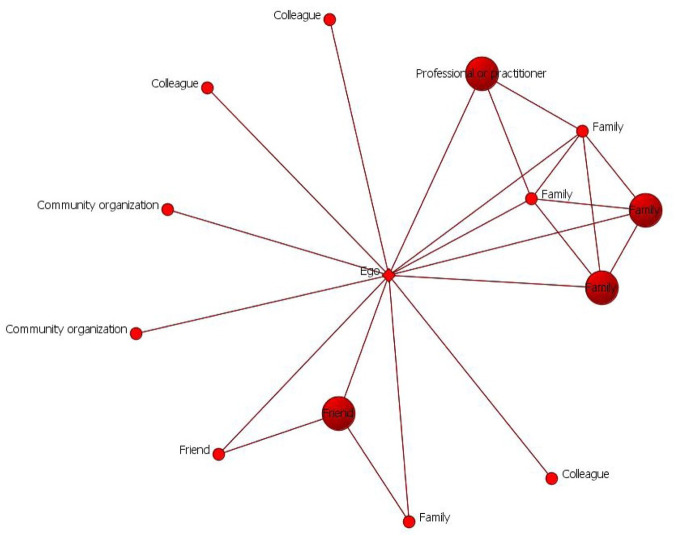
A participant’s social network.

The first step was a content analysis of the following elements: (1) individuals mentioned according to the types of tie as reported by the participants (family; friends; romantic partners; health professionals (e.g. psychologists, social workers, or therapists); managers or colleagues; teachers, staff or classmates; and community organizations); (2) types of support mentioned categorized based on Barrera’s (1981) and Carpentier and White’s (2001) classifications (emotional, instrumental, social-related, informational, and companionship); (3) direction of the support (given or received); and (4) interconnection (ties between network members). This information was entered into an adjacency matrix, that is, a table where all actors mentioned by ego (including ego) are placed in rows and columns, and in which binary values (0–1) indicate the presence or absence of ties. We generated an egocentric sociogram of each participant’s PPSN using the ORA-LITE software (Carley, 2014). Figure 1 shows an example of a participant who mentioned four people in his PPSN.

Three variables were calculated on this pilot sample: the size of the personal network (number of people mentioned), the PPSN’s density (number of actual ties divided by number of total ties, excluding ego from calculation), and the proportion of bidirectional support ties. The latter was calculated by dividing the number of ties from which at least one type of support was received or provided by the total number of ties. Thus, alter–alter ties are not directional, and reciprocity refers only to support exchanges between ego-alters. We conducted association tests (Student’s *t* test and analysis of variance (ANOVA)) with these three variables, along with age and gender. We also conducted nonparametric tests given the small sample size in each group, but results did not differ significantly from those obtained with parametric tests.

### Reflexive thematic qualitative analysis

**Figure 2.**
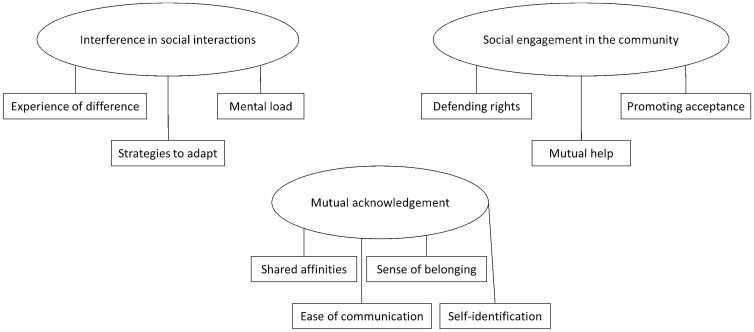
Identified themes and subthemes.

The second step was to qualitatively explore the main themes regarding types of support shared among autistic peers and the relational contexts in which support was given or received. Throughout the recruitment and interview process, the first author (IC) has been journaling. She noted her impressions, observations, and recurring themes. After the 31st interview, a saturation in qualitative data appeared to be reached, which justified the end of the data collection phase (Charmaz, 2006). The interviews were entirely transcribed, except for personally identifiable information. Of the 31 participants, 21 reported receiving, providing, or sharing support with at least one autistic peer. Based on this qualitative dataset (including all ties mentioned by these 21 participants), a reflexive thematic analysis (TA) grounded in an experiential framework was conducted, as we aimed to capture the perspective of autistic adults on their support networks, with a particular focus on the meaning they attributed to peer support (Braun & Clarke, 2022). We chose reflective TA for its flexibility, enabling us to conduct research from a qualitative experiential framework based on a social constructivist theoretical approach (Berger & Luckmann, 1967) with an abductive analytical orientation. We followed the six phases of the reflexive TA to develop, analyze, and interpret patterns across the qualitative dataset in an iterative process. After familiarizing ourselves with the dataset (1), interviews were coded one after another to produce semantic codes (2), and the codes were then grouped to generate initial themes (3). We noted the connections between them in the participants’ discourse and existing knowledge about social experiences, autism, and social support (4). The themes were refined by comparing the coded excerpts with the entire dataset. They were subsequently analyzed and more precisely defined (5) until the writing of this article (6). This reflexive TA recognizes the value of “subjective, situated, aware and questioning researchers” and their role as situated interpreters (Braun & Clarke, 2022, p. 5) of participants’ ideas or meanings shared about social support. As mentioned in Community Involvement, the coding and analysis process benefited from the interweaving of our different positionalities. Collective coding enhances understanding, interpretation, and reflexibility. Discussions between the authors took place at each step of the analysis process to clarify the themes and to validate each other’s interpretations with a complementary objective. Data processed in the two-step analysis were compared through triangulation to deepen our understanding of what peer support represents in the participants’ everyday life. Finally, as shown in Figure 2, we identified three themes under the topic of peer support: (1) interference in social interactions, (2) mutual acknowledgment, and (3) social engagement in the community.

## Results

### Personal support networks: size, composition, density, and bidirectionality

Overall, participants described their networks as ranging from 13 to 79 people. As shown in Table 1, no significant difference was found between men and women. However, the mean size of personal networks among participants in the 19 to 24 age group was significantly smaller than that of older participants (25–45 years old). Most participants reported having autistic or neurodiverse people in their network, or people living with another condition or mental health issues—“small issues of something” as described by Melody (25 years old). Twenty-one of them have identified autistic peers in their social network. However, only two participants identified these people as part of their PPSN. Friends and family members held an important place in the participants’ PPSN. The participants’ PPSN was predominantly composed of friends, ranging from 0% to 100% with a mean of 41%. Family members accounted for 0% to 80% (*M* = 39%). For example, Melody underlined that “the first people we [her and her brother, both autistic adults] refer to are our parents.” Psychosocial and healthcare professionals comprised 0% to 40% (*M* = 8%), and a spouse or romantic partner represented 0% to 25% (*M* = 7%). Community organization ties, which include people with whom the participants engage in interest or recreational groups (e.g. sports teams, theater groups, life-size role-playing games, organizations, associations and Facebook groups), also appeared, but to a lesser extent (0%–40%; *M* = 4%). Only one person mentioned a work colleague among the people in their PPSN (0%–20%; *M* = 0.6%).

**Table 1.** Network size, PPSN’s density, and bidirectionality indexes.

	*n*	*M*	Median	Associations
Network size				
Men	14	40.57	39.00	*t*(29) = 0.82, *p* = .420
Women	17	35.88	33.00	
19-24 years	10	28.10	27.00	*F*(2, 13) = 6.16, *p* = .013 19-24 < 25-45
25-45 years	13	43.31	44.00	
46 years and older	7	43.00	35.00	
PPSN’s density				
Men	14	0.39	0.32	*t*(29) = –1.26, *p* = .217
Women	17	0.51	0.40	
19-24 years	10	0.37	0.32	*F*(2, 27) = 0.72, *p* = .497
25-45 years	13	0.46	0.40	
46 years and older	7	0.53	0.50	
PPSN’s bidirectionality				
Men	14	0.51	0.60	*t*(29) = 2.27, *p* = .031
Women	17	0.71	0.80	
19-24 years	10	0.58	0.60	*F*(2, 27) = 0.18, *p* = .835
25-45 years	13	0.64	0.67	
46 years and older	7	0.66	0.80	

Some participants identified fewer than five “important” people making up their PPSN (two participants mentioned four people and one participant mentioned three). Like Hazel (31 years old), some participants stated that it was not the size of the social network that was important, but rather the strength of the ties: “Having a bigger circle . . . everyone tells you that it’s really cool, but then you eventually realize that it’s really not cool . . . So my three friends, honestly, it’s perfect, I don’t want more.” The average density index of the PPSNs is of 0.45, suggesting that slightly less than half of the alters were connected to each other according to ego. This observation is confirmed by the analysis of the egocentric sociograms of the PPSN generated for each participant. The average density index was slightly smaller in men and younger participants (19–24 years old), but these differences were not statistically significant. Regarding shared support in the PPSN, the average bidirectionality of ties appeared to be approximately 62% (see Table 1). The mean bidirectionality indexes were higher in women and older participants, although only the former association was statistically significant.

### Personal support networks by type of support: companionship, emotional, informational, instrumental, and social-related support

**Figure 3.**
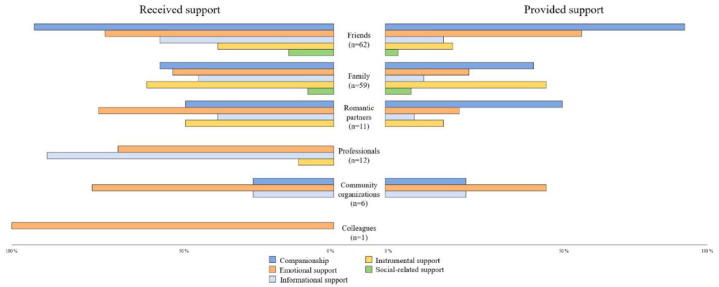
Support exchanges according to the type of relationship and the type of support.

Although support was shared in more than half of the cases, Figure 3 shows differences regarding the support that was received or given in the PPSN for different types of ties and support.

Those providing companionship support offer opportunities for social outings, accompany the person during recreational activities, and create enjoyable moments (Barrera, 1981). In our sample, it was perceived and presented as shared with friends, family members, the spouse or romantic partner, and people from community organizations.

Emotional support, which was mainly shared with friends, family members, the spouse or romantic partner, and people from community organizations, was received to a greater extent than was given. Emotional support involves experiences of intimacy, companionship, demonstrations of tenderness and approval, and the fulfillment of needs of love, self-esteem, and trust. These interactions are manifested in conversations where one confides in someone and shares private matters (Barrera, 1981). A participant mentioned, for example, that he “met a friend who helped [him] a lot to defend [himself] and deal with anxiety” (Walter, 23 years old). We observed the same trends for informational support, except for community organization ties, where support was given and received to the same extent.

Informational support designates advice and directive guidance to solve practical problems in everyday life (Carpentier & White, 2001) and was mainly provided by professionals and friends. It includes giving information. For example, a participant described a friend this way: “She’s my friend and my social guide. Let’s say that I have something delicate to say to someone, I call her, and she tells me how to say it” (Kyla, 27 years old).

Instrumental support has a concrete character and includes various types of practical assistance in everyday life, such as transportation, help for purchases or moving, and financial assistance. In our sample, it was mainly shared among friends, family members, and the spouse or romantic partner. It was also received to a greater extent than it was given. For example, Melinda (50 years old) confided that she had “a good husband” and that “if he weren’t there, I couldn’t fulfill my needs due to my small salary.”

Finally, social-related support encompasses socialization aspects (Carpentier & White, 2001). It includes people who foster connections or facilitate ego relationships with others, providing recommendations from professionals or introductions to a group of friends. In our sample, participants mainly referred to this in relation to their relationships with friends and family members. Social-related support was often combined with companionship or informational support, as shown in the following example: “My partner comes with me to my medical appointments. It helps me to have information” (Fabrice, 40 years old).

Overall, the participants’ PPSN, consisting of the three to five people they consider most important in their everyday life, is composed of rich and multiplex ties associated with the different types of shared support, the diversity of relationship types, and the mean density of their PPSN. Support among autistic peers was only rarely present in the participants’ most proximal network.

### Support among autistic peers: same types and important but “different”

Compared with the quantitative data describing the participants’ PPSN, the qualitative analysis focused particularly on peer support. The different types of support shared by autistic peers, even when they were not part of participants’ PPSN, were described as important by them. They were also perceived as different compared with those provided by others in their circle.

Several participants mentioned sharing emotional support with autistic peers, combined with the exchange of advice and tips to solve certain everyday life problems. Although several said that they confided in their closest friends, they sought support and advice from autistic people outside of their PPSN in cases of crisis, sensory overload, or other autism-related aspects. Kyla explained, “Since she is ASD and she went through the same things, sometimes I tell her: ‘This happened to me, what would you do [in my place]?’”

Shared social-related support was also mentioned, such as connecting people in their network or sharing resources, references, and help for diagnostic assessments or support procedures. Here are two examples:

I told them: “You need to meet. You both have autistic and gifted kids, and you are neighbours.” . . . Since then, they have seen each other! (Laura, 47 years old)

There was one guy who was looking for a place . . . I told him: “I went to [name of the assessment centre], they were really good. If you need a paper from the doctor, if you want a diagnosis, you can go there.” (Derek, 36 years old)

Melody added, however, that the support of her peers does not always replace the help of a professional:

Yesterday, I talked with someone who is autistic. He also has depression . . . He tried to help me, but, you know, he didn’t have the capacity and the knowledge to give me the help that a psychologist or a social worker could give me. (Melody, 25 years old)

Regarding companionship, participants said that they went out with other autistic people (e.g. go to a restaurant, see a show, go to a museum or a cinema). These activities could be organized by associations, as explained by Derek: “They did a singles’ evening for autistics . . . It was really fun. There also was an Aspie pride dinner that they did, and another organized a hike. . . . It was really cool.” Thus, groups for autistic people appeared to be favorable spaces to connect with other autistic people. Like Laura, participants indicated that they met new acquaintances in these groups:

There are people whom I met in groups and with whom I continue to have discussions [on Facebook]. Without necessarily considering them as close friends, but still, [they are] people with whom it’s nice to come back to some subjects. (Laura, 47 years old)

For Derek, as for others, groups fulfilled a need to be listened to, supported, and socialized. They were also an opportunity to share tips and advice: “We live similar situations on the social level. There are some who say: ‘You know, I do this in this situation, and it works.’” (Derek). For Hazel, it is a weekly opportunity to “settle down” and “be with people like me!” Most participants appreciated peer support groups. However, they could also lead to disappointment, as explained by Kyla: “I don’t go there anymore because I had shutdowns after every meeting. We talked about stuff that didn’t interest me, and everyone cut each other off. It irritated me a lot . . . I did not receive anything in return.” James (35 years old) also expressed disappointment because the conversation topics did not focus on autism and how it was lived on a daily basis: “I wanted to talk about autism . . . They were like, ‘Hey, did you see this movie?’”

Last, there were few mentions of instrumental support between autistic peers. Participants were indeed more likely to seek instrumental support from their parents, romantic partner, and close friends.

### Interference in social interactions: experience of difference, strategies to adapt and mental load

When discussing peer support, all 21 participants mentioned negative experiences related to their daily social relationships, highlighting the specific characteristics of peer support. To understand these characteristics (discussed in the next section), it is necessary to understand the difficulties mentioned. A recurrent aspect was the idea and the feeling of being different, whether in the way they reacted, behaved, thought, or functioned. All of them mentioned various situations of interference in face-to-face interactions. Some said that they had difficulty understanding undertones and implicit or second-degree messages, which would often cause discomfort, misunderstandings, and sometimes conflict with others. Some participants also mentioned a mismatch between what they said and their nonverbal expressions, leading others to poorly decode their message. James (35 years old) explains,

For example, if I say, “I am feeling very bad,” she is going to say to herself, “Well, he doesn’t feel that bad.” Because I don’t look that bad . . . my sentence does not give the impression that it’s really serious, whereas what I am saying is: “I am really feeling bad, I have suicidal thoughts.”

Aware of their “difference” and the challenges that it causes in their social interactions with allistic people, some participants tried to learn as much as possible about social interaction codes, to “get out of [their] comfort zone” (Hazel) and do “constant work” (Derek). The cognitive effort associated with social interactions was continuous and energy consuming. Like Hazel, some participants reported self-feedback practices of “overanalyzing” the social interaction in which they had just participated to react better in the future: “You go through it again, and then you have your plan in action. You analyze all the possible outcomes” (Hazel). These repeated cognitive and emotional efforts can lead to a state of hypervigilance and, over time, exhaustion. In that respect, Kyla stressed, “I’m always overchecking myself, and then at one point when I’m too tired, well, then the valve breaks and I end up saying something that shouldn’t be said.” Despite this mental load, the desire to connect with others remains present, as explained by Derek, who described, “pushing [himself] to socialize.” Later in the interview, he added that “it’s a human need; some say that autistics don’t care about others and that they don’t need to socialize. It’s not true . . . It’s as important as for others” (Derek). In sum, interference in social interactions with allistic individuals appears crucial to better understanding the meanings that participants attributed, by way of comparison, to their relationships with autistic peers.

### Mutual acknowledgment: affinities, communication, belonging, and self-identification

Peer support, when mentioned, was described as based on shared affinities and a greater ease of communication. When two autistic people get along, the feeling of connection in the relationship was described by some as more intense and developing faster than with other people. Laura explained, “With autistic people, when it connects, it connects strongly, it connects quickly, we quickly arrive at the intense and then in the sharing of very personal things.” Derek and James indicated that it is easier to understand and talk with autistic people. In this regard, they said,

Everyone respects the bubble of everyone . . . So, since everyone is [autistic], it’s easy being several people together. (Derek, 36 years old)

When people talk, I’m always saying to myself: “But what do they want to say?” . . . And then, I was at [non-profit organization for autism] and I realized that . . . wow, it’s so light all of a sudden, it’s really easy. They talk, and I understand everything; I don’t feel like there is something behind it. (James, 35 years old)

Some participants also found it easier to interact with allistic people who are familiar with the realities of autism and those who also experience social differentiation, such as those belonging to social minority groups, whether racialized or neurodivergent.

In addition, a sense of belonging to the “autistic” group emerged from the participants’ discourse. The period following the official diagnosis was described as a pivotal moment that contributed to self-identification as an “Autistic,” “Aspie,” or “autistic woman.” Moreover, some specified that they had “recognized” themselves or have been recognized by others, such as friends, acquaintances, or a parent, before starting their assessment process, as underlined by Derek: “One of my neighbours told me: ‘You really have autistic traits. Did you ever think about it?’” Participants repeatedly related their personal experiences to those of other autistic people, as shown, among other things, by their frequent use of the pronouns “we” and “us.”

### Social engagement in the community: rights, mutual help, and acceptance

This mutual acknowledgment process led participants to become socially involved in their communities. These types of commitments revolved around defending rights, promoting autism awareness and acceptance, and mutual aid. The creation of self-help structures and the organization of recreational activities or workgroups to put pressure on universities and employers were mentioned. Two participants explained the projects they had in mind:

I have a project to set up a work committee at the union to handle accessibility issues, not only for autism. . . . I talked about it to a colleague . . . a wheelchair user, and tried to find people with different disabilities and accessibility needs. (Laura, 47 years old)

I’ve talked about a [housing] cooperative . . . Because the noise, the light, all the hypersensitive things . . . If we could go further and get support . . . [without] creating a world apart. If we can just help each other, all the best. It’s not about isolating ourselves from society, it’s only about making places where we feel good. (Derek, 36 years old)

Regarding raising awareness and acceptance among the general population and in intervention and autism research settings, some participants mentioned talking openly about their diagnosis with the people they met, organizing conferences about autism, and participating in studies about autism. In short, shared peer support fosters the sharing of experiences, such as the interference experienced in social interactions, and contributes to a sense of belonging to the autistic community. These elements can lead to social engagement practices, which, in turn, become valuable sources of support within the community.

## Discussion

This research is rooted in a social constructivist framework that acknowledges that social reality is shaped by social meanings and that its interpretation always depends on individuals’ socially and locally situated viewpoint (Berger & Luckmann, 1967). Consequently, we adopted an approach that is sensitive to the meaning actors give to their experiences of social relationships. We analyzed the composition of the proximal personal support network (PPSN) of 31 autistic adults in Québec and the place occupied in their egocentric network by shared support among autistic peers. The PPSN is part of the participants’ personal network, which varies greatly in size, ranging from 13 to 79 people. The group comprising participants between 19 and 24 years old had a significantly smaller mean size for the personal network compared with older participants. However, a larger number of people in one’s egocentric network does not necessarily ensure better support, hence the relevance of analyzing the PPSN. The average density of the PPSN was 0.45. In other words, about half of the possible connections between the members of the network were present. Indexes were lower for men and younger participants (19–24 years old), but these differences were not statistically significant. The PPSN was predominantly composed of friends (41%) and family members (39%), who mostly provided companionship, emotional support, instrumental support, and informational support. These means resemble those found by Lei, Brosnan, et al. (2020) in their study on the networks of young autistic adults entering university, reporting PPSNs made up of friends (49%–59%) and family members (27%–39%). The significant presence of friendship relationships in PPSNs challenges the still prevalent belief that autistic people do not have friends, which is corroborated by Mendelson et al. (2016) meta-analysis. Moreover, the fact that most ties (62%) in the PPSNs were described as bidirectional also challenges the representation of autistic people simply as people needing support to which those around them respond. In this study, women reported significantly higher average bidirectionality, which could be partly explained by the fact that they mentioned professionals less often than men (bidirectional ties are less socially expected in that type of relationship). As McGhee Hassrick et al. (2020), we found that women’s egocentric networks have fewer family members, but appear to have more interconnected actors in this study. It should be noted that the analysis of support ties only used the ego-alter ties, identified as important by the ego. Nevertheless, the results reveal more complex realities, wherein personal networks and PPSNs are made up of frequent multiplex and bidirectional exchanges.

Most participants referred to autistic peers (*n* = 21). Although peers provided support, participants rarely identified them as being part of their proximal personal support network (*n* = 2). However, the qualitative results underline the importance of peer support for several participants. For example, the types of emotional and informational support sought or provided among peers differed from support exchanged with other people in their network, often taking the form of empathetic listening and advice related to the specific social experience of being autistic. Shared affinities and interests were also perceived as facilitating connections with others and friendships, in line with other studies (Elmose, 2020; Sosnowy et al., 2019). Besides being perceived as unique, interactions with other autistic people were described as simpler and communication easier. As in other studies, participants indicated feeling a greater sense of mutual understanding and acceptance when interacting with other autistic people, without excluding the possibility of also experiencing this feeling with allistic people (Botha et al., 2022; Crompton, Hallett, et al., 2020; Elmose, 2020; Milton & Sims, 2016). In addition, several studies support the finding of greater reciprocity or sharing in social interactions among autistic people (Chen et al., 2021; Crompton, Sharp, et al., 2020; Milton & Sims, 2016), which may further promote sharing of support among peers. Similar to other qualitative studies, participants sought people who did not negatively judge their “difference,” whether they were autistic or not (Milton & Sims, 2016; Sosnowy et al., 2019).

This study reveals the ties between autistic peers, although they are mostly not characterized by proximity. These kinds of “weak ties” (Granovetter, 1973) appeared in the participants’ narratives as being especially valuable during times of crisis, when one needs to access resources that are further away than those available in the immediate environment, such as during the period surrounding the diagnosis or when seeking professional support to recover from a health issue. However, these ties between autistic peers, who are not part of the PPSN, provide specific types of support, such as emotional (e.g. opening up on their difference without feeling judged), informational (e.g. sharing tips in social situations), and social-related support (e.g. peer recommendations of specialized resources). In this study, although weak ties are less redundant and, as such, provide access to important information, it seems like participants’ “weak” ties also provide some of the resources normally expected of “strong” ties, like emotional support. Torres (2019) proposed the term of “elastic ties” to refer to nonstrong and nonweak relations between people who spend hours each day and share intimate details of their lives with those whom they do not consider “confidants.” Nonetheless, they provide each other with the support and practical assistance typically seen in strong-tie relationships. A single social tie can vary between strong and weak depending on the social situation and many “fall outside weak and strong; they are elastic in allowing elders (and other marginal groups) to connect and secure informal support while maintaining their distance and preserving their autonomy” (Torres, 2019, p. 235). It is reminiscent of what we found in our research, where participants shared information at crucial moments of their personal path in relation to autism and a sense of belonging to a specific social group (i.e. the neurodivergent community), which led to the exchange of significant emotional support, despite these peers not being considered as friends or close ones by the participants.

Therefore, the “cohesive power of weak ties” (Granovetter, 1973, p. 1360), contained in the concept of “elastic ties” (Torres, 2019), emerges from the participants’ discourse concerning their sense of belonging to the autistic group and the (mutual) acknowledgment process, which ultimately fosters social engagement within the community. Similar to the findings in Botha et al.’s (2022) study, participants in our research also reported associating with other autistic people or minority groups to assert their rights and resist social stigmatization. Moreover, social engagement was demonstrated through selective participation in research studies focused on issues deemed important by the participants and potentially beneficial to other autistic people in the community (Botha et al., 2022). It is important to emphasize the significant history of engagement by, and for autistic people, and the support among peers within the autistic and neurodivergent communities, which has led to the establishment of online communities through dedicated websites, special events (e.g. Autscape), and rights advocacy groups (e.g. Autistic Self Advocacy Network, Autism Network International; Kapp, 2020).

According to Small (2017), people are generally much more willing to confide in people they do not know than what they tend to say or what social science theories predict about how people use their personal network. Most people confide in their closer ones, but they also share very personal worries, problems, and concerns with others, such as gyms or online gaming partners, coworkers, baristas, therapists or people at church. However, the current study reveals that the way of interacting (e.g. communication modalities, body position, gestures, gaze), contexts (e.g. in an environment protected from sensory microaggressions) and discussion topics (e.g. concerns, interests, autism and neurodivergence, seeking formal services) are central conditions for these relationships for participants. According to them, these conditions are rarely met with their relatives or other nonautistic relations. Thus, the particularities of relationships and shared support among autistic peers must be understood in conjunction with the interference, tensions, and discomfort experienced in social interactions, especially with allistic or nonautistic people. Milton’s proposition regarding the “double empathy problem” offers a way to better understand these feelings. Milton postulates that the greater the gap between the dispositions of two actors involved in an interaction, the more significant the breakdown in communicational reciprocity (Milton, 2012). In this respect, the dispositional differences between allistic and autistic people would cause misunderstandings on both sides. In other words, allistic people would have as much difficulty understanding autistic people as autistic people have in understanding allistic people (Chown, 2014; Milton, 2012). On the one hand, due to their status as a minority group, autistic people are—willingly and unwillingly—compelled to familiarize themselves with the codes governing social interactions of the majority group composed of allistic people, whereas the reverse does not necessarily apply (Chown, 2014; Milton, 2012). On the other hand, participants reported finding it easier to interact with allistic people who are familiar with neurodiverse realities or those who belong to social minority groups. This finding suggests that the social experience of difference, neurological or not, is an important source of experiential knowledge, which can generate solidarity bounds between individuals and socially minorized groups.

## Conclusion

To this day, there have been few studies that address the support networks of autistic adults that take into account the meanings that autistic people attribute to peer support in their network. This study shows the relevance of using mixed methods research that includes analysis on both social network structure and qualitative aspects (Lei et al., 2025). In this study, we aimed to understand the subjective perceptions that autistic adults have about their proximal personal support network and the meanings they attribute to shared support among autistic peers. The results reveal the significant role of peer support in the lives of the participants, even though they may not necessarily be considered as part of their proximal personal support network. This type of support differs from what is available in their immediate environment, highlighting its unique importance and impact. However, it is important to note that the limit of five people included in the PPSN could have reduced the likelihood of autistic people being named as important alters.

This study has several limitations that should be acknowledged. First, given the sample size and its voluntary composition, we cannot determine whether it represents or not the experience of the majority of autistic people and their experiences with peer support. Regarding the pilot sample used in the quantitative dataset, the preliminary results obtained may be confirmed later with a statistically powered sample. Second, interviews were conducted with participants who used verbal language. The current findings may not be generalizable to all autistic people. Therefore, this research could be replicated using an adapted protocol (oral or written) to reach participants with diverse communication preferences and cognitive functioning, for example, by using visual aids, breaking down tasks, giving simple instructions or questions, and organizing several meetings rather than a single interview. As we observed in our protocol, the support of an interpreter, a trusted person, or a caregiver is a facilitating factor. Accessibility in research is an important issue as these groups are too often under-represented in autism research, meaning that their experiences of the social world are still largely unacknowledged. Third, the current paper focuses on the allistic/autistic division, but other aspects could influence the quality of ties, and the types of support exchanged. Some differences were noted regarding gender and age, but cultural background might be an important aspect. For example, the ways in which support manifests itself could vary according to family values. Finally, despite rigorous data processing and extensive efforts of data triangulation and co-interpretation, it is still possible that some aspects of the participants’ experience have been overlooked.

To conclude, considering that personal networks are dynamic and that ties evolve over time, it would be relevant to develop a longitudinal design to further explore the concept of “elastic ties” in the context of autism. It would also be interesting to investigate how these networks change throughout different life stages and significant life events, such as the transition to adulthood, parenthood, and older adulthood. This pilot sample shows promising results and suggests research directions that warrant further investigation. It additionally serves as an invitation to reflect on the contribution of autistic people’s firsthand accounts of their experiences and the relevance of the network analysis approach in studying support and quality of life in autistic people.

## Supplemental Material

sj-docx-1-aut-10.1177_13623613251369908 – Supplemental material for Nor weak ties, nor strong ties: Personal support networks and relations between autistic peers Supplemental material, sj-docx-1-aut-10.1177_13623613251369908 for Nor weak ties, nor strong ties: Personal support networks and relations between autistic peers by Isabelle Courcy, Noémie Cusson and Nuria Jeanneret in Autism

## Notes

1. The neurodiversity paradigm describes autism as one of many existing human neurological variants (Pellicano & den Houting, 2022; Singer, 1998). Adopting an affirmative stance, it emphasizes the use of identity categories such as “Autistic,” “people on the spectrum,” and “neurodiverse people.”

2. “Allistic is an empowering term that reframes autism and autistic traits as a difference instead of an abnormality” (Monk et al., 2022, p. 792)
