# Supplementary material for: Nor weak ties, nor strong ties: Personal support networks and relations between autistic peers
Source: Autism. 2025 Sep 9;30(1):97–110. doi: 10.1177/13623613251369908 (PMC12717286; doi:10.1177/13623613251369908)
Supplement: sj-docx-1-aut-10.1177_13623613251369908 – Supplemental material for Nor weak ties, nor strong ties: Personal support networks and relations between autistic peers [file sj-docx-1-aut-10.1177_13623613251369908.docx]

**Network Questionnaire**

1. **Personal Network**

I would like to know more about your personal social network, including the main people you have interacted with, talked to, or done activities with over the past six months. This could include, for example, your partner, roommate, parents, siblings, coworkers, friends, autistic friends, or any other person.

For each person mentioned in different spheres (family, friends, acquaintances, professionals):

1. What is your relationship with this person?
2. How long have you known this person?
3. How often do you see or talk to this person?
4. Does this person live far from you?
5. What do you do together?
6. Is there any dissatisfaction with this relationship, or does it suit you?
7. **Support Network**

Suppose you need advice or guidance on something, whether a specific or general topic, for example, job searching, moving, or managing finances.

- Whom would you ask? About what topic?
- Can you describe a situation or give an example of where this happened?

If you feel like doing an activity, such as going for a walk, going to the movies, having a coffee, or playing computer games, is there someone you do it with?

- With whom? For which activity?

Suppose you need help with small tasks, a service, or borrowing something. Would you ask someone?

- Whom would you ask?
- Can you describe a situation where this happened?

Do you feel close enough to someone to confide in them about important, very personal matters?

- Who?
- Can you describe a situation or give an example of where this happened?

1. **Reciprocity**

Among the people mentioned, in the past six months, have you given advice to someone?

- To whom?
- Can you describe what happened?
- Have you given advice to anyone else in the past six months?

Among the people mentioned, in the past six months, have you helped someone with small tasks, a service, lending money, or lending items?

- To whom?
- Can you describe what happened?
- Have you helped anyone else in the past six months?

Among the people mentioned, in the past six months, has anyone confided in you?

- Who?
- Can you describe what happened?
- Have other people confided in you in the past six months?

1. **Interconnections** (*ties between network members*)

Among all the people you mentioned, who knows whom?

- Among those who know each other, who gets along well?
- Do you do activities together?

1. **Proximal personal support network (PPSN)**

In your opinion, who are the five most important people in your life today?
